# Clinical opportunities in combining immunotherapy with radiation therapy

**DOI:** 10.3389/fonc.2012.00169

**Published:** 2012-11-26

**Authors:** Steven E. Finkelstein, Mayer Fishman

**Affiliations:** ^1^21st Century Oncology Translational Research ConsortiumScottsdale, AZ, USA; ^2^Department of Genitourinary Oncology, H Lee Moffitt Cancer CenterTampa, FL, USA

**Keywords:** dendritic cells, immunotherapy, radiation effects, stereotactic radiosurgery, immune modulation

## Abstract

Preclinical work in murine models suggests that local radiotherapy plus intratumoral syngeneic dendritic cells (DC) injection can mediate immunologic tumor eradication. Radiotherapy affects the immune response to cancer, besides the direct impact on the tumor cells, and other ways to coordinate immune modulation with radiotherapy have been explored. We review here the potential for immune-mediated anticancer activity of radiation on tumors. This can be mediated by differential antigen acquisition and presentation by DC, through changes of lymphocytes’ activation, and changes of tumor susceptibility to immune clearance. Recent work has implemented the combination of external beam radiation therapy (EBRT) with intratumoral injection of DC. This included a pilot study of coordinated intraprostatic, autologous DC injection together with radiation therapy with five HLA-A2(+) subjects with high-risk, localized prostate cancer; the protocol used androgen suppression, EBRT (25 fractions, 45 Gy), DC injections after fractions 5, 15, and 25, and then interstitial radioactive implant. Another was a phase II trial using neo-adjuvant apoptosis-inducing EBRT plus intra-tumoral DC in soft tissue sarcoma, to test if this would increase immune activity toward soft tissue sarcoma associated antigens. In the future, radiation therapy approaches designed to optimize immune stimulation at the level of DC, lymphocytes, tumor and stroma effects could be evaluated specifically in clinical trials.

## INTRODUCTION

### RADIATION EFFECTS

A conventional view of radiation is an immune attenuator. In this perspective, damage, and destruction are the effects on living tissues – whether they are tumor, normal stroma, and parenchyma, or leukocytes. In the medical application of therapeutic radiation, this is a measured induction of apoptosis and other cell death within a carefully defined volume. The impact of radiation on leukocytes can be viewed in similarly detrimental terms, whether attenuating lymphocyte numbers as tolerable side effect (Johnke et al., 2005; Lissoni et al., 2005) a therapeutic effect, such as part of an allogeneic transplant protocol (Wei et al., 2004; Gupta et al., 2011), or precipitating a secondary malignancy (Brill et al., 1962). The measurement of accumulated radiation injuries, such as micronuclei and DNA breakage in circulating lymphocytes, has been proposed as a direct assay of individuals’ relative radiosensitivity (Minicucci et al., 2005; Tang et al., 2008; Ishihara et al., 2012); that sensitivity can be relevant to either toxicity or to treatment efficacy.

**FIGURE 1:**
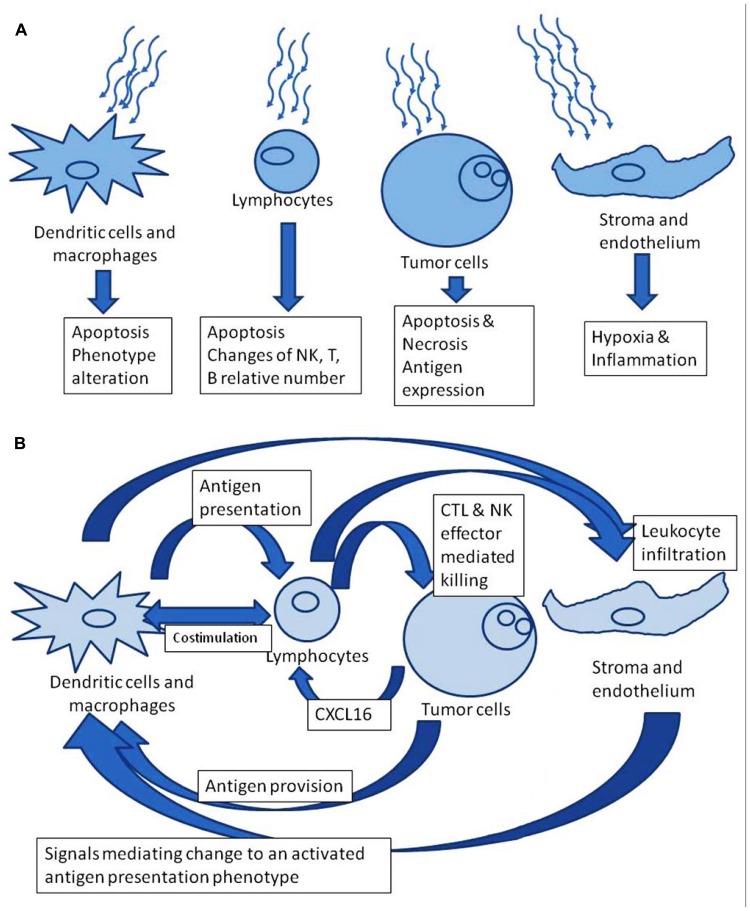
**(A)** Radiation effects in isolation. **(B)** Downstream theoretically favorable immune modulation after irradiation.

We focus here on the effect of radiation on the bilateral relationship of tumor with the immune system, not just on the effects of radiation on the tumor or on the leukocytes, separately. Considered in isolation, radiation to any particular cell could be anticipated to have a detrimental impact. However, there is an opportunity in the interplay of tumor cell death, induced antigen expression on tumor cells, and inflammatory signals from the irradiated volume which affect lymphocyte and dendritic cell (DC) activation. **Figure 1** contrasts the perspectives of isolated versus system effects of irradiation. Immunotherapeutic impacts can be coordinated with therapeutic tumor irradiation. In this way, the whole therapeutic effect can exceed the sum of its parts.

### PROCESSES OF CELLULAR IMMUNITY

Physiologic process of antigen presentation and lymphocyte activation are complex processes, and subject to modulation because of the tumor microenvironment (Fricke and Gabrilovich, 2006). Immature myeloid cells acquire antigen, whether by vaccination or through phagocytosis of material in the tumor microenvironment. These cells then mature, with acquisition of cell surface proteins such as MHC class I and II on which peptides derived from the antigen source can be presented, to interact with particular antigen-specific idiotypic receptors on T lymphocytes (discussed, for example, by Liao et al., 2004). Other maturational markers such as CD80, CD86 facilitate costimulation interactions, particularly the process of activation versus tolerogenic influence on those lymphocytes (these illustrated in Topalian et al., 2012, where the focus is on the PD-1/PDL-1 interaction, for example). The interaction of lymphocytes with the antigen-presenting cells, occurs in lymph nodes to which the DC migrate as part of the maturation process, and the subsequent potential anticancer effect of lymphocytes then is a consequence of lymphocytes’ expansion within the lymph node, circulation, and penetration into the tumor mass. Other lymphocyte pathways, such as natural killer (NK) cells, may be influenced by T cell activation and the tumor microenvironment, but do not require specific education and costimulation by DCs. Other antigen-presenting cells, such as macrophages, and inflammatory cells such as neutrophils may influence the tumor microenvironment (Fricke and Gabrilovich, 2006) in a way that indirectly, but overwhelmingly alters the polarization of macrophages, DC, or the activation state effector lymphocytes. Overall, the potential effect of radiation on the preponderance or phenotype of many cell types, some of which are discussed below, could influence availability of tumor antigens, the acquisition of the antigens by immature antigen-presenting cells, the migration of those cells to lymph nodes, the eventual polarization into tolerogenic or immunogenic phenotype, the efficiency of interaction with lymphocytes, the stimuli leading to intratumoral migration of lymphocytes, the extent of activation of the lymphocytes that are within the tumor, and the susceptibility of (still living) tumor cells to immune lysis. As for many anticancer pharmaceutical interventions, we are only beginning to understand the influences that irradiation can effect on this system.

## RADIATION EFFECTS IN ISOLATION

### RADIATION EFFECTS: THE TUMOR

The fundamental mechanism of tumor regression following radiotherapy is by induction of DNA damage in the neoplastic cells. This accumulation of DNA breaks and consequent insufficient repair is the trigger for pathways including Bcl2 family apoptotic and anti-apoptotic proteins, p53-dependent, and independent pathways, or TRAIL [tumor necrosis factor (TNF)-related apoptosis-inducing ligand) dependent mechanisms (Maduro et al., 2008; Roos and Kaina, 2012). However, this basic view is still not a complete picture of microenvironmental changes within tumor-associated endothelial cells, inflammatory infiltrates, or of systemic responses to the tumor. Areas of higher dose exposure, for example adjacent to brachytherapy seeds, or at hot-spots inside the bulk of the tumor may have markedly different pathways to cell death, emphasizing necrotic mechanisms not apoptotic ones (Nagorsen et al., 2003; Overwijk et al., 2003; Finkelstein et al., 2004; Klebanoff et al., 2004; Kakinuma et al., 2007). Additionally, the time course of changes of antigen expression by the irradiated cells may be relevant, with different patterns that are dependent on radiotherapy techniques’ dose-rate and energy level (Finkelstein et al., 2011).

Besides the phenomenon of cells dying within an irradiated tumor, several processes have specific relevance to immunotherapy. Some relate to inflammation and clearance of antigens within the irradiated volume. Of the most interest are the processes that influence acquisition of a more activated general immune phenotype or of a more activated tumor-specific immune phenotype. The most dramatic clinical outcome is when a distant tumor mass regresses, the abscopal effect. Clinical examples described as case reports (Kingsley, 1975; Postow et al., 2012; Stamell et al., 2012) and preclinical examples are discussed in more detail below. Less apparent outcomes, still with major clinical impact, may occur as well. These include accelerating or completing definitive clearance of the tumor which was being irradiated. Another important impact can be clearance of other metastatic disease that was not clinically apparent because it was microscopic; this could lead to prevention of systemic recurrence as a consequence of radiation-triggered immune activation in the primary tumor.

Moravan et al. (2011) describe persistent inflammatory changes consisting of neutrophil and T cell infiltrates, within brains of C57BL/6 mice, as a specific and lasting effect of irradiation, in the absence of tumor. The protein CXCL16 (CXC motif ligand 16) is released from irradiated tumor. This binds the CXCR6 receptor, found on activated effector T cells (Matsumura and Demaria, 2010). A murine model, including use of a CXCR6 knockout control mouse, demonstrated this mechanism of T cell infiltration to the tumor (Matsumura et al., 2008). Another group, surveying 63 cytokines, found that CXCL16 levels went down after 30 Gy irradiation of skin (not tumor) in a murine model (Xiao et al., 2013). The specific relevance in clinical use remains to be elucidated.

High mobility group box 1 (HMGB1) is a protein which is released from some dying cells, including tumor cells killed by anthracyclines (Fucikova et al., 2011), and in a hyperthermia and radiation combination model (Schildkopf et al., 2010 ), and with radiation and chemotherapy combination treatments for colorectal cancer cell lines, particularly with the combination (Frey et al., 2012). The HMGB1 effect on DC can include maturation and a chronic inflammatory state (Fucikova et al., 2011). It is an important question whether a clinically relevant (adverse) changes of DC phenotype (Popovic et al., 2006 ) or of downstream T cell effector activity (Liu et al., 2011) occur from tumor therapy-derived HMGB1. It is not clear if irradiation protocols leading to higher or lower systemic HMGB1 levels would be better for induction of a general anticancer immunophenotype.

In a clinical report on patients receiving primary, curative-intent fractionated external beam radiation therapy (EBRT) for prostate cancer, Hurwitz et al. (2010) describe observation of consistent systemic changes. These were increases of (systemic) levels of tumor-derived protein Hsp72 (heat shock protein), and of inflammatory cytokines IL-6 and TNF-α. Circulating CD8^+^ T cells and NK cells showed increases of 2.1- and 3.2-fold, respectively. While the changes of these particular proteins or leukocytes do not directly prove a functional augmentation of the systemic antitumor response, they are illustrative of impacts on the host’s overall immunophenotype because of events within the tumor.

### RADIATION EFFECTS: THE LYMPHOCYTES

There is not significant systemic lymphopenia from prostate cancer EBRT, our group has observed (Finkelstein et al., 2012d). Others suggest that hypofractionated radiation therapy can mediate a decrease in CD4^+^ and CD8^+^ lymphocyte number, but not of NK and of B lymphocytes. This effect was counterbalanced in those patients receiving combined androgen blockade, with goserelin and flutamide, suggesting a converse effect of testosterone suppression (Johnke et al., 2005). In a report describing serial flow cytometry analyses lymphocytes of cervical cancer patients (stage IIB through IVA) being treated with larger field external beam irradiation and concomitant intracavitary brachytherapy again it was observed that total lymphocyte count went down. In the patients without progressive disease, the CD8^+^ T cell and NK cell percentages increased. The authors commented that these increases are consistent with a role of CD8^+^ T cell and NK cell in definitive tumor clearance (Lissoni et al., 2005). This is comparable with the CXCL16 mechanism discussed above (Matsumura et al., 2008).

Brachytherapy is a radiation therapy modality with markedly different kinetics of radiation exposure. In brachytherapy seed placement (Iodine 125 or Palladium 103) or radioembolization with Yttrium 90 microspheres (Carr and Metes, 2012), there is a longer exposure to radiation than with conventional external beam treatment, with potential for most of the circulating blood volume be transiently in very close proximity of the radioactive source. Carr and Metes (2012) evaluated the impact on lymphocytes of Yttrium 90 embolization of hepatocellular cancer, with finding that there was an early decrease on T cell number (both CD4^+^ and CD8^+^) and B cell number (assayed by CD19), but not on NK cells or neutrophils. Over time, the deficits persisted significantly for some patients; an impaired recovery was associated with worse prognosis. This could reflect a disease impact on the lymphocyte repopulation, more so than an ongoing radio-isotope mediated suppression (Lissoni et al., 2005).

### RADIATION EFFECTS: THE DENDRITIC CELLS

The tumor microenvironment has potential to modulate the phenotype of DC to favor the pathologic tolerance of the tumor (Fricke and Gabrilovich, 2006). The focus of the therapeutic rationale for placing DC into the tumor microenvironment (discussed below) is that radiation will alter that effect, but the impact of radiation onto DC should be considered separately. Isolating the issue, higher doses of radiation (25–30 Gy) than would be used in a standard fractionated radiotherapy plan (generally less than about 2 Gy), were studied in an experimental setting assaying *ex vivo* priming of DC by Cao et al. (2004), in a report with a focus on multiple sclerosis patients. They report that the irradiated DC would still stimulate T cell proliferation in the MLR (mixed lymphocyte reaction) assay but at a lower level, and with higher T cell production of IL-2 and IL-4. Phenotypic changes related to maturational markers were observed, with lower levels of CD80 (B7.1), CD86 (B7.2), and HLA-DR on the DC.

On the other hand, Jahns et al. (2011) studied *ex vivo* preparations of leukocytes, focusing on quantitative functional impact on DC versus the impact onto lymphocytes. They found that DC are less sensitive to apoptosis than lymphocytes, and maintained the same functional level (in terms of cytokine profiles, surface markers, and maturation) after a radiation dose that impaired T cell function. In particular, there was lower expression of DC maturational markers (CD80, CD86, and HLA-DR) and the T cells had less activation. Bogdándi et al. (2010) tested splenocytes of mice (C57BL/6) exposed to increasing doses of radiation, up to 2 Gy, with the most sensitivity for B cells (at 2 Gy), but more resistance in the NK cells, DC and regulatory T cells, thus observing a similar pattern of relative sensitivity to irradiation. The specific impact of acquisition or suppression of these DC maturational markers on clinical outcomes must be studied empirically to address whether the net change was favorable.

Liao et al. (2004) isolated the issue of irradiation of DC, again in a model system with C57BL/6 mice, with B16 melanoma. The loading of the DC was by transfection with adenovirus engineered to express the MART-1 antigen, termed AdVMART1; the B16 melanoma expresses the MART-1 antigen, as do the majority of human melanoma specimens. Murine DC were obtained from bone marrow (femur and tibia), and cultured and transfected *in vitro*, after which they express the (full length, human) hMART-1 protein, and also the immunodominant MART-1_27-35_ peptide. The DC irradiation protocol consisted of 10 Gy, in a single fraction in just over 2 min. To assay the effect of irradiation of the DC on the class 1 antigen-presentation process, DC culture was irradiated (or not treated), then (immediately) transfected with AdvMART1, then injected into (non-tumor bearing) mice; this was repeated at a 7 days’ interval. Then after an interval of 10–14 days, the T lymphocytes from the spleen were assayed with the finding that acquisition of elevated level of T lymphocytes with specificity for the test antigen (MART-1_27-35_ peptide) was eliminated by the radiation protocol. Similarly, subsequent challenge to test mice with B16 melanoma injection showed protection only for un-irradiated DC treatment, but not for mice not injected with DC, and not for mice injected with DC that had been treated on the irradiation protocol. Further, they investigated the potential maturation-related mechanisms for irradiation of DC affecting the capacity or tendency to present the class I epitopes of MART1; they observed that maturational markers of DC (particularly CD80, CD86, and MHC class I and II) were not changed. In testing the response to CD40L and interferon gamma (IFN-γ) stimulation (maturational signals), although there was (pretreatment) a decrease of some maturational markers (CD80, CD83, MHC class II), after treatment, the difference was not observed. Looking at those results, the effect of DC irradiation appears to be neutral or suppressive (Liao et al., 2004).

In a next set of investigations, to test for antigen-presentation effects isolated from antigen processing, a modified DC/tumor system was used. The HLA-A2.1/K^b^ transgenic mice bear human HLA-A2; the modified tumor B-16A2/K^b^ does as well. When DC from these mice were prepared and treated as above, but then instead of being transduced with the adenovirus, the DC were instead pulsed with the immunodominant MART-1_27-35_ peptide. These DC (or control DC that were pulsed but had not been irradiated) were use to vaccinate mice; 10 days after the last vaccination the mice were challenged with B-16A2/K^b^ tumor, it was found that mice in the group treated with the DC that had been irradiated had better survival, and a higher induced immunity as measured by IFN-γ production in an ELISPOT assay with the MART-1_27-35_ peptide (Liao et al., 2004). Thus, the irradiation of DC with 10 Gy in this model system, where antigen processing and maturation were not much changed or a little worse, showed a *better* anticancer effect, attributed to improved presentation.

## THE TUMOR MICROENVIRONMENT

### LOCAL IMMUNE SUPPRESSION

The immune system in the cancer-bearing host cancer has defects that allow the tumor cells to evade clearance. The way that immune privilege is maintained is heterogeneous across different disease stages and patients. Some characterizations can be in terms of DC phenotype; an excess of myeloid-derived suppressor cells (MDSC) that are not mature DC, but rather suppress DC function to impair anticancer immunity (Almand et al., 2000). Other characterization can focus on the tumor microenvironment. That kind of suppression can be observed to operate through elaboration of particular proteins which have receptors on DC and MDSC, in some models and some clinical examples. Those microenvironment derived molecules include vascular endothelial growth factor (VEGF), tumor growth factor β (TGF-β), reactive oxygen species, the enzyme indoleamine-2,3-deoxygenase, granulocyte-macrophage colony stimulating factor (GM-CSF), interleukin-8, interleukin-10 (reviewed by Fricke and Gabrilovich, 2006). Specific inhibition of these pathways can have a favorable impact on DC phenotype and the capacity for meaningful immunologically mediated anticancer response, for example a murine tumor model was induced to be immunologically rejected by use of VEGF depleting antibody (Gabrilovich et al., 1999); a clinical trial using sequential bevacizumab (humanized anti-VEGF antibody, Roche USA, Indianapolis, IN) and then low dose subcutaneous IL-2 did not demonstrate a significant clinical impact nor impact on DC phenotype for VEGF depletion (Finkelstein et al., 2010). However, in a clinical trial utilizing another VEGF chelation strategy, with a similar testing scheme, found no functional improvement as a consequence of ziv-aflibercept treatment (formerly “aflibercept,” also called VEGF-trap; Sanofi-Aventis, Bridgewater, NJ). Changes that were observable as flow cytometry defined phenotypic changes of DC from patients following treatment, however, were favorable (Fricke et al., 2007).

### RATIONAL PLACEMENT OF DC VERSUS RADIATION THERAPY TIMING

Almost any radiation therapy protocol can be analyzed with respect to its theoretical immune impact, either on an anatomic or temporal perspective. From an anatomic perspective, regions of the treatment target volume with the highest doses could be anticipated to have higher and faster peaks of tumor cell death, and availability of antigenic material. Regions of lower dose could have radiation induced changes of antigen expression on the tumor cells. Leukocytes and stroma also would respond to irradiation, with variable amounts of induced regional inflammatory cytokines, or penetration with other inflammatory cells, such as macrophages and neutrophils. Since DC can be anticipated to potentially become activated when placed into this environment, that is a key rationale for intratumoral, versus intravenous or subcutaneous administration.

Considering a temporal perspective, the best time to introduce DC into an irradiated tumor is much less clearly defined. The onset of inflammatory changes may have a significant latency, particularly in conventionally fractionated treatment plans, with a high number of treatment fractions in the 180–200 cGy range. Placement of DC too early or too late could result in their exposure to a microenvironment more resembling an intact (immunosuppressive) tumor. The onset of apoptosis or other cell death, or changes of antigen expression on the tumors themselves is more difficult to predict in clinical tumors – when would DC have the richest supply? The potential that injected DC themselves would be irradiated, after acquiring antigen, but before migration out to lymph nodes also must be considered. The migration time appears relatively fast (on the order of a couple of days), but as Liao et al. (2004) found, the possibility of enhanced antigen presentation after DC irradiation is another theoretically favorable consideration.

### INTRODUCTION OF DC INTO THE TUMOR LOCALE

Nikitina and Gabrilovich (2001) initially described the basic model of intratumoral DC injection coordinated with sub-curative irradiation of the primary tumor, in a model system using methA sarcoma (in Balb/C mice) and C3 tumor (in C57BL/6 female mice) tumors. Key findings for the combination treatment group (but not for the monotherapies or untreated controls) were longer survival of the mice, with higher T cell titer of tumor-specific tetramer peptides, and higher CD8 T cell response to tumor-specific peptides. Additionally DCs obtained from spleens of syngeneic mice and marked with fluorescent tracer that were injected subcutaneously were demonstrated to track into the irradiated tumor. Further, the T cell-mediated immunity was sufficient to reject tumor rechallenge. In sum, the unmanipulated DC that were placed into irradiated tumor-mediated systemic, lasting antitumor immunity, without any other systemic modulation (Nikitina and Gabrilovich, 2001).

In another murine tumor system (C57BL/6 female mice with the D5 tumor, which is a poorly immunogenic subclone the B16-BL6 melanoma, and with the MCA205 fibrosarcoma), Teitz-Tennenbaum et al. (2003) observed superior survival in mice treated with a combined radiation and intratumoral DC injection protocol. Further they found that loading of the DC with antigen *in situ* was superior to *ex vivo *loading with irradiated tumor lysate. This contributes to support the idea of particular microenvironmental attributes of the irradiated tumor that mediate the changes on DC function and the consequent antitumor immune effect (Teitz-Tennenbaum et al., 2003). In further work with the D5 tumor, they found that the loading and presentation of D5-associated antigens by DC was enhanced by D5 irradiation, independent of the low level of tumor cell death that was directly induced by radiation. Finally, trafficking of DC to regional tumors was better after tumor irradiation (Teitz-Tennenbaum et al., 2008), consistent with the findings of the earlier report discussed above (Nikitina and Gabrilovich, 2001). On the other hand, assays for several inflammatory cytokines (using cultures of tumor cells), including IL-12^p70^, TNF-α, IFN-γ, IL-6, and IL-10 did not show changes following the tumor irradiation, and tumor-specific CD8^+^ T cells did not accumulate in the tumor (Teitz-Tennenbaum et al., 2008).

## CLINICAL TRIALS OF RADIATION PLUS DENDRITIC CELLS

### INTRATUMORAL DC INJECTION

Several groups have developed clinical trials toward a goal of more effective anticancer immune response by tumor irradiation coordinated with intratumoral placement of DC. Primary radiation therapy for treatment of clinically localized prostate cancer was studied in a pilot trial, by our group (Finkelstein et al., 2012a). While the technique of intraprostatic injection was described generations ago, in a canine model addressing therapy of benign hypertrophy (O’Conor and Ladd, 1936), this is the initial trial of intraprostatic injection of apheresis derived autologous DC. There are several features of the clinical scenario that could be favorable. These include the expectation that the local therapy could be definitive, the accessibility for an injection technique that can be standardized, and simultaneous use of androgen suppression, which may favor an increased capacity for immune response (Windmill and Lee, 1999; Johnke et al., 2005). Further, the bulk of residual (metastatic, extraprostatic) disease should be microscopic, at worst, in well-selected patients, and should have a multi-year latency until detectable recurrence, which could allowing time for immune clearance to go to completion. Disadvantages of this system, conversely, are that no immediate therapeutic effect is discernible. By limiting the inclusion to individuals with HLA-A*0201 haplotypes, it was hoped that it would thus be feasible to use an immunological endpoint to give a readout of an acquisition of a higher titer-specific CD8^+^ CTL. To this end, serial assays of the titer of T lymphocytes by response to stimulation with class 1-associated peptides were used with the ELISpot (enzyme-linked immunosorbent spot-forming) IFN-γ assay. This endpoint tested for specificity to the peptides, derived from PSA, PSMA, PAP, Her2/neu, and p53, representing prostate-associated and prostate cancer-associated proteins (Finkelstein et al., 2012a).

Inclusion required localized cancers, without radiologically identified metastasis, but with high-risk features (T-stage, PSA, Gleason score) for eventual recurrence. The five patients were treated with a conventional therapy schedule of 28 months’ androgen suppression, 45 cGy EBRT over 25 fractions, which was then followed by brachytherapy seed placement. Autologous DC were prepared from a single pretreatment apheresis, and injected after the 5th, 15th, and 25th radiation therapy fraction, in each case on a Friday, so as to give the injected DC about 72 h to potentially migrate out from the radiotherapy field, before the next (6th or 16th) fraction on the following Monday. Overall, the apheresis and injections were well tolerated. Some patients had detectable increases of titers for some of the peptides, but persisting elevations were not apparent. The low number of patients, and the heterogeneity of disease features, precludes a meaningful long-term efficacy assessment (Finkelstein et al., 2012a).

A second trial developed in our group addressed combined neo-adjuvant apoptosis-inducing EBRT plus intratumoral DC injection in larger group of patients, with soft tissue sarcoma (STS) diagnoses. The immunologic objective was to test for detectable increase of T lymphocyte titer on testing with autologous STS tumor cell lysate, using an ELISPOT assay (Finkelstein et al., 2012b). Patients with clinical stage T2N0M0 high-grade STS of the extremity, trunk, or chest wall were treated with standard neo-adjuvant EBRT 5040 cGy in 28 fractions of 180 cGy coordinated with additional DC injection, after weeks 2, 3, and 4. The DC were prepared from a pretreatment apheresis, *ex vivo* expansion and culture, and given as intratumoral injection of 10 million DC.

Secondary analyses included functional T cell activity, toxicity tabulation, primary tumor responses, and analysis of DC migration to lymph nodes, *in vivo. *Seventeen patients completed neo-adjuvant EBRT with and DC injection. Fifty-two per cent showed anti-autologous tumor cell immune responses, as determined using pre- and post-treatment ELISpot assays (Finkelstein et al., 2012b). This titer increased after the last DC injection.

Additionally, chromium release assays revealed that after the treatment there was a statistically significant improvement of the functional cell-killing response to autologous STS lysate. Examination of the tumor from the post-radiation, definitive-intent surgery showed that the combination treatment was associated with a dramatic accumulation of intratumoral T cells. Presence of CD4^+^ T cells in the tumor positively correlated with tumor-specific immune responses that developed following combined therapy. Accumulation of MDSC but not of regulatory T cells negatively correlated with the development of tumor-specific immune responses.

The treatment was well tolerated, with no toxicity higher than grade 2 was observed during combined DC/EBRT. Post-operative wound complications were observed in five of the 17 patients (29%), applying the NCIC criteria of a secondary operation for wound repair or wound management without secondary operation. Twelve of 17 patients (71%) were progression free after 1 year.

Image-guided visualization of cellular-based vaccine migration was demonstrated for each patient. Experiments with ^111^In labeled DCs demonstrated that these antigen-presenting cells need at least 48 h to start to migrate from tumor site (Finkelstein et al., 2012b). This experience led to a multi-institutional trial which is currently accruing (Finkelstein et al., 2012c).

## CONCLUSION

The coming years offer opportunities to transform the phenomenon of radiotherapy-induced anticancer immune response from isolated case reports into a predictable therapeutic goal. To this end, several components and perspectives must be unified and coordinated. One is the understanding of how to use systemic therapies to make the host lymphocyte compartment and antigen-presenting cell compartments be primed for stimulation. Some examples of immune modulators with the potential to be having a significant impact on the phenotypes of the DC compartment include TLR9 agonists (Brody et al., 2010; Kim et al., 2012; Zhang et al., 2012) all *trans* retinoic acid (Mirza et al., 2006), inhibitors of VEGF, TGF-β, or use of other cytokines (Antony et al., 2005; Charo et al., 2005; Gattinoni et al., 2005; Klebanoff et al., 2005, 2011; Zeng et al., 2005; Seung et al., 2012). Comparably, stimulation of the lymphocyte compartment with checkpoint inhibitors and cytokines also appears poised to make a significant contribution to clinical practice. It will be of interest to see if radiation therapy can be systematically used to advantage in combinations with those new agents as well.

Another component will be the ways to provide tumor-associated antigen to the immune system. While recombinant vaccines and tumor lysates and synthetic peptides have attributes of convenience and definable antigen sets, they cannot be considered interchangeable with tumor irradiation as a source. Unique features of tumor irradiation include simultaneous elaboration of subtle microenvironmental changes with the capacity to improve antigen presentation, total tumor as a source of antigen, elaboration of radiation-induced antigens, and provision of antigen even before or independent of radiation-induced cell kill. Further, evolving flexibility of radiation technique, particularly in relation to conventional fractionation, hypofractionation, brachytherapy, stereotactic radiosurgery techniques, and high intratumoral dose exposure may be particularly of interest for optimization of antigen production and repolarization of the tumor microenvironment. The best way for radiation to trigger an abscopal response may be related to tumor effect, DC effect, lymphocyte effects, or indirect modulation of the way the tumor is affecting leukocyte compartments.

A third component of interest is cellular therapy, particularly intratumoral DC injection – many questions about timing with respect to irradiation, details of *ex vivo* preparation remain to be addressed empirically. Optimal host preparation, patient selection, and antigen loading could improve outcomes as well. The best volume and number of injected DC merits empiric study. Finally, as a necessary part of clinical development, there must be some focus on specific diagnoses.

In summary, it is clear that radiation is a modulator of the interaction of the tumor and immune compartments, and their relationships with each other. Careful study of the microenvironment of the irradiated tumor should lead to exciting opportunities for putatively localized anticancer treatments to be leveraged to make the irradiated tumor a catalyst for systemic anticancer response.

## Conflict of Interest Statement

The authors declare that the research was conducted in the absence of any commercial or financial relationships that could be construed as a potential conflict of interest.
